# Stationary gaze entropy predicts lane departure events in sleep-deprived drivers

**DOI:** 10.1038/s41598-018-20588-7

**Published:** 2018-02-02

**Authors:** Brook A. Shiferaw, Luke A. Downey, Justine Westlake, Bronwyn Stevens, Shantha M. W. Rajaratnam, David J. Berlowitz, Phillip Swann, Mark E. Howard

**Affiliations:** 1 Centre for Human Psychopharmacology, Swinburne University of Technology, Hawthorn, Australia; 2 Institute for Breathing and Sleep, Austin Health, Melbourne, Australia; 3 Monash Institute of Cognitive and Clinical Neurosciences and School of Psychological Sciences, Monash University, Clayton, VIC Australia; 4 University of Melbourne, Melbourne, Australia; 5 VicRoads, Kew, Australia

## Abstract

Performance decrement associated with sleep deprivation is a leading contributor to traffic accidents and fatalities. While current research has focused on eye blink parameters as physiological indicators of driver drowsiness, little is understood of how gaze behaviour alters as a result of sleep deprivation. In particular, the effect of sleep deprivation on gaze entropy has not been previously examined. In this randomised, repeated measures study, 9 (4 male, 5 female) healthy participants completed two driving sessions in a fully instrumented vehicle (1 after a night of sleep deprivation and 1 after normal sleep) on a closed track, during which eye movement activity and lane departure events were recorded. Following sleep deprivation, the rate of fixations reduced while blink rate and duration as well as saccade amplitude increased. In addition, stationary and transition entropy of gaze also increased following sleep deprivation as well as with amount of time driven. An increase in stationary gaze entropy in particular was associated with higher odds of a lane departure event occurrence. These results highlight how fatigue induced by sleep deprivation and time-on-task effects can impair drivers’ visual awareness through disruption of gaze distribution and scanning patterns.

## Introduction

An estimated 20–30% of all fatal road accidents are attributable to driver fatigue^1^. Drowsiness resulting from reduced or impaired sleep, or driving during the body’s circadian nadir (usually night time) are the main causes of driver fatigue, and are particularly prevalent among shift workers and individuals with sleep disorders^2,3^. Fatigue can also arise from prolonged engagement in tasks requiring sustained attention such as long distance driving^4,5^. Drowsiness causally contributes to traffic accidents by impairing cognitive processes and physical responses that are essential for driving^6–9^. There is currently a lack of reliable objective measures to ascertain the level of drowsiness-related impairment, which remains a barrier to implementation of strategies to reduce associated driving risks^3^.

Visual perception is the primary mode through which drivers monitor their environment^10^; making the selection and processing of visual information essential cognitive functions for driving. Impairment in active visual scanning of the road and surrounding environment is associated with reduced hazard perception and higher risks of road crashes^11–13^. In recent years, blink parameters have been investigated as possible physiological indicators of driver drowsiness in simulated^14,15^ and real world^16,17^ driving settings. While eyelid closure characteristics may indicate increased sleepiness and correlate with decrements in driving performance^18^, they fall short in providing further insight into how sleep deprivation may alter visual perception and attention (i.e. when eyes are open) to increase erroneous driving incidents. Therefore, examining changes in overall visual scanning behaviour may further elucidate how sleep deprivation affects driving performance by altering visual perception and attention.

Visual scanning or gaze behaviour refers to how overt attention is spatially distributed for the purpose of sampling visual information through interspersed fixations occurring in-between saccadic eye movements^19^. The distribution of gaze is governed by interconnected attentional networks including sensory selection, oculomotor control and working memory^20,21^. In naturalistic viewing environments, gaze control also involves higher-level processes to mediate the interaction between top-down assessment of specific task requirements and bottom-up response to stimuli saliency^22–24^. Such capacity to recruit a breadth of networks and cognitive processes makes gaze a relevant behavioural output to utilise for examining the overall neurocognitive influences of sleep deprivation on visual scanning, which is essential for driving performance.

Gaze entropy (measured in *bits*) refers to quantitative approaches that have been used to assess visual scanning behaviour during engagement in tasks with high visuospatial demand such as driving^25^, simulated aviation^26–28^ and surgical procedures^29^. The concept of entropy used in these methods is that of information theory, which describes the amount of required information to generate a given sequence as a measure of overall uncertainty^30^. Based on such concept, stationary gaze entropy (*H*_*s*_) is a method in which Shannon’s entropy equation (1) is applied to the probability distribution of fixation coordinates to calculate the average level of uncertainty in the spatial distribution of a sequence of fixations generated in a given timeframe^31^. Higher entropy or uncertainty, in this case, indicates a wider distribution of fixations across the visual field, suggesting greater dispersion of gaze^29^.

Another method, gaze transition entropy (*H*_*t*_), builds on the stationary distribution of fixations to further examine patterns in visual scanning. To achieve this, the conditional entropy equation (2) is applied to Markov chain matrices^32^ of fixation transitions. This provides an average measure of predictability of visual scanning patterns where higher entropy suggests less structured or a more random pattern of scanning behaviour^31,33^. Together, stationary and transition gaze entropy measures provide a quantitative method of characterizing visual scanning behaviour in naturalistic environments. An increase in *H*_*s*_ has been reported in association with higher surgical task load, while *H*_*t*_ appears to increase with elevated anxiety^28^ and secondary cognitive load^34^ during simulated aviation tasks. These findings suggest that top-down interference on gaze control may lead to disturbance of visual scanning as overall gaze distribution becomes more dispersed and transition patterns less structured. Since higher cognitive functions which facilitate top-down control and other relevant processes such as attention and working memory are susceptible to the effects of sleep deprivation^35,36^, both *H*_*s*_ and *H*_*t*_ may be similarly altered in sleep-deprived drivers.

The present study aimed to explore how overall gaze behaviour is affected by sleep deprivation and task-induced fatigue during an on-road driving task with a natural visual environment, and whether decline in driving performance can be predicted by driving duration and changes in gaze behaviour. To this end, the impact of 1-night total sleep deprivation on fixation and blink parameters, saccade amplitude, gaze entropy (*H*_*s*_*, H*_*t*_), and driving performance was investigated during 2-hours of real driving on a closed track. It was hypothesised that sleep deprivation would reduce fixation rate while increasing blinking rate, blink durations, saccade amplitude and gaze entropy, and that these effects would increase across the 2-hour driving duration as participants become more fatigued due to extended engagement with the task. It was also expected that more lane departure events would occur in the sleep-deprived condition, gradually increasing with driving duration; and that the changes in ocular measures would predict the likelihood of a lane departure event occurring.

## Results

### Subjective sleepiness

Participants had 7.7 ± 0.49 SD hours of sleep the night before driving in the rested condition, and had no sleep the night prior to driving in the sleep-deprived condition. Using the Karolinska Sleepiness Scale (KSS), participants reported a higher level of sleepiness (*t*_[8]_ = 7.84, *p* < 0.001) in the sleep-deprived condition (7.56 ± 1.01) than in the rested condition (3.22 ± 1.56).

### Condition effect

**Figure 1:**
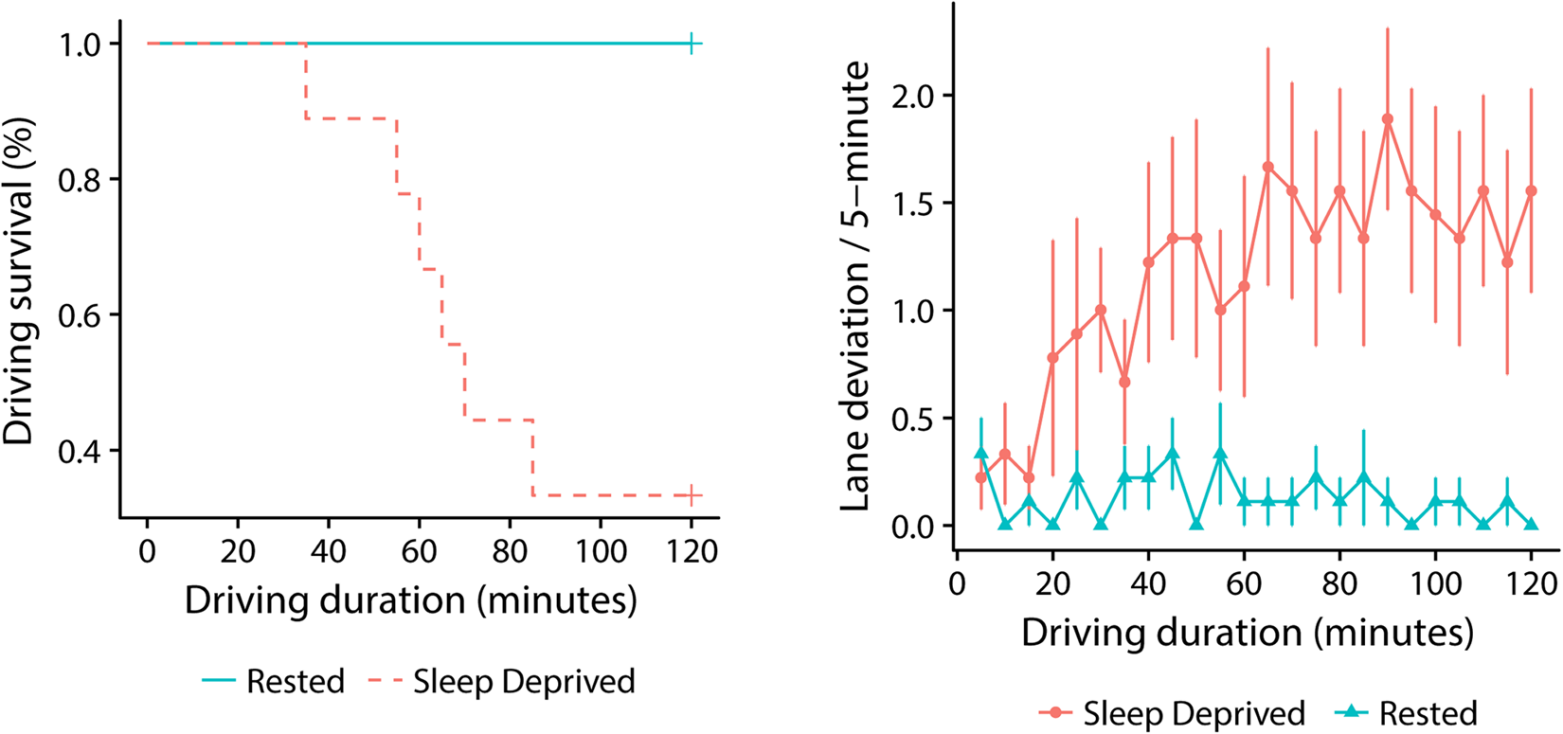
Kaplan-Meier survival curve for the six early terminations of driving in the sleep-deprived condition, depicted in red line (*Left*). Number of lane departure events per 5-minute blocks of driving duration with standard error for sleep-deprived and rested conditions (*Right*).

Pre-drive reaction time in Psychomotor Vigilance Task (PVT)^37^ was slower (*t*_[8]_ = 5.54, *p* = 0.001) in the sleep-deprived (279.78 ms ± 58.95 SD) than the rested (225.39 ms ± 44.51 SD) condition. Blink rate (*F*_[1,8]_ = 7.04, *p* = 0.029) blink duration (*F*_[1,8]_ = 5.93, *p* = 0.041), saccade amplitude (*F*_[1,8]_ = 6.87, *p* = 0.031), stationary gaze entropy (*F*_[1,8]_ = 7.00, *p* = 0.030) and gaze transition entropy (*F*_[1,8]_ = 7.11, *p* = 0.029) increased in the sleep-deprived condition while fixation rate reduced (*F*_[1,8]_ = 19.39, *p* = 0.002). There was no significant effect for fixation duration, so it was not considered for further analysis – see Table 1 for details. Mean driving duration reduced from 120 minutes in the rested condition to 77.22 ± 34.73 SD following sleep deprivation (*p* = 0.002) as six of the nine participants (66.67%) terminated the drive early – see Fig. 1 (*Left*). Sleep deprivation also increased overall rate of lane departure events (rate ratio = 3.79, 95% CI: 2.53, 5.85, *p* < 0.001)-see Fig. 1 (*Right*).

**Table 1:** Mean values and standard deviations of ocular and entropy variables in rested and sleep-deprived conditions, with *F* statistics and significance level.

	Mean ± SD Rested	Mean ± SD Sleep Deprived	*F*_[1, 8]_	*p*
Blink rate (count/m)	39.42 ± 14.73	52.40 ± 19.89	7.04	**0.029**
Blink duration (ms)	179.88 ± 24.89	200.75 ± 21.86	5.93	**0.041**
Fixation rate (count/m)	76.91 ± 16.50	60.80 ± 19.74	19.39	**0.002**
Fixation duration (ms)	561.38 ± 108.02	510.27 ± 113.00	1.69	0.232
Saccade amplitude (°)	19.16 ± 11.05	27.77 ± 13.26	6.87	**0.031**
*H*_*s*_ (bits/m)	0.001 ± 0.0001	0.003 ± 0.002	7.00	**0.030**
*H*_*t*_ (bits/m)	0.006 ± 0.0004	0.011 ± 0.006	7.11	**0.029**

### Condition by driving duration effects

**Figure 2:**
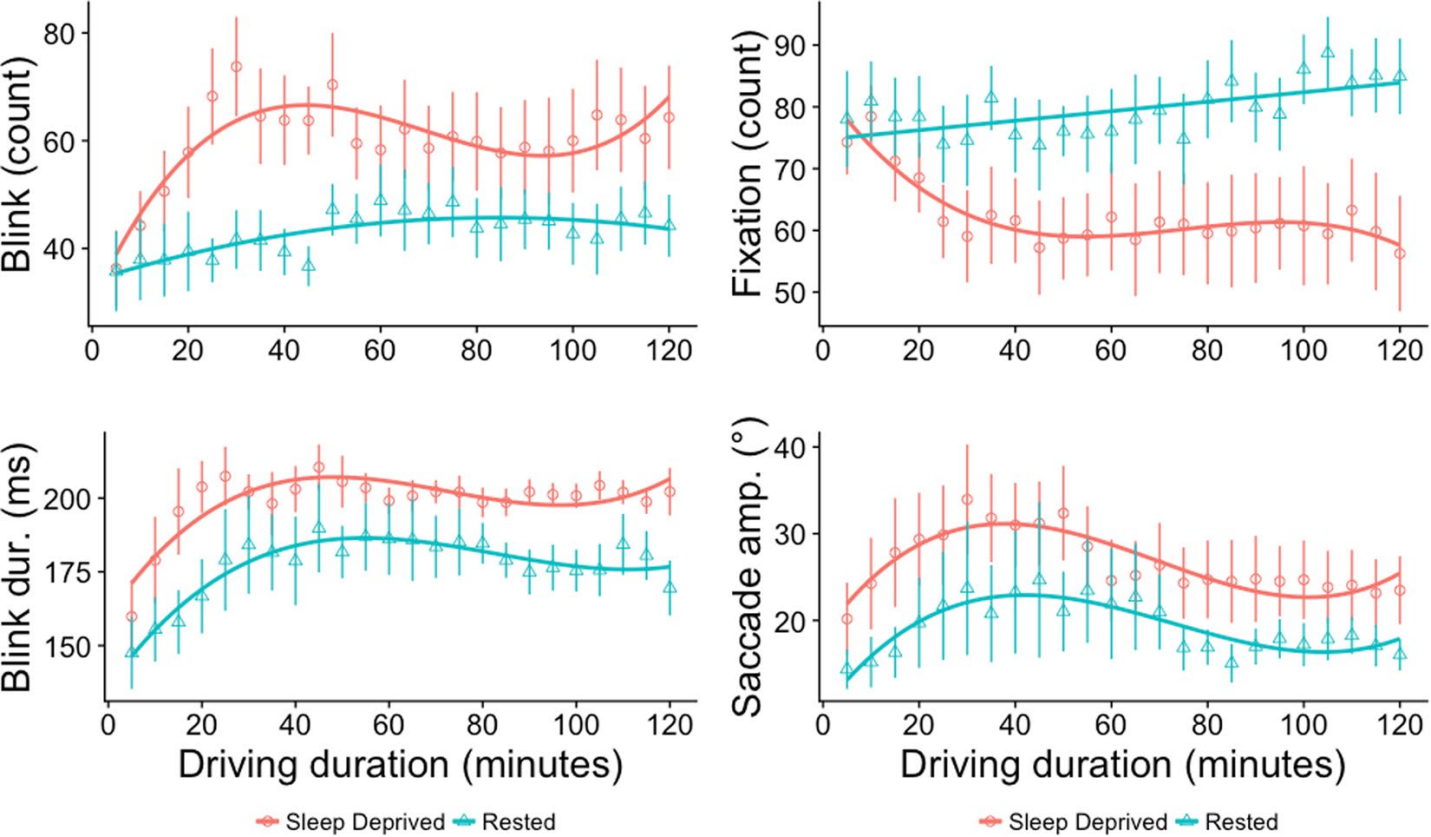
Mean and standard errors across driving duration by condition (red = sleep-deprived, green = rested) with curvilinear fits for blink rate -Top Left, sleep-deprived: (*ŷ* = 30.727 + 1.964*x* − 0.033*x*^*2*^ + 0.0002*x*^*3*^), rested: (*ŷ* = 41.780 + 0.277*x* − 0.002*x*^*2*^); blink duration-Bottom Left, sleep-deprived: (*ŷ* = 168.606 + 2.332*x* − 0.036*x*^*2*^ + 0.0002*x*^*3*^), rested: (*ŷ* = 136.400 + 2.332*x* − 0.030*x*^*2*^ + 0.0001*x*^*3*^), fixation rate - Top Right, sleep-deprived: (*ŷ* = 55.829 − 1.090*x* − 0.016*x*^*2*^ − 0.0001*x*^*3*^), rested: (*ŷ* = 63.081 + 0.077*x*); and saccade amplitude - Bottom Right, sleep-deprived: (*ŷ* = 33.462 + 0.769*x* − 0.014*x*^*2*^ − 0.0001*x*^*3*^), rested: (*ŷ* = 14.878 + 0.723*x* − 0.012*x*^*2*^ + 0.0001*x*^*3*^).

The combined effects of sleep deprivation and driving duration on blink rate (*F*_[23,408]_ = 66.25, *p* < 0.001, *R*^2^ = 0.78), blink duration (*F*_[23,408]_ = 30.61, *p* < 0.001, *R*^2^ = 0.61), fixation rate (*F*_[23,408]_ = 64.35, *p* < 0.001, *R*^2^ = 0.77) and saccade amplitude (*F*_[23,408]_ = 55.15, *p* < 0.001, *R*^2^ = 0.74) were best fitted with polynomial curvilinear regression models. In the sleep-deprived condition, blink rate (*ŷ* = 30.727 + 1.964*x* − 0.033*x*^*2*^ + 0.0002*x*^*3*^), blink duration (*ŷ* = 168.606 + 2.332*x* − 0.036*x*^*2*^ + 0.0002*x*^*3*^), fixation rate (*ŷ* = 55.829 − 1.090*x* − 0.016*x*^*2*^ − 0.0001*x*^*3*^) and saccade amplitude (*ŷ* = 33.462 + 0.769*x* − 0.014*x*^*2*^ − 0.0001*x*^*3*^) showed some fluctuations across the driving period as they were best fitted with a cubic function. Similar patterns of alteration were observed in the rested condition for blink duration (*ŷ* = 136.400 + 2.332*x* − 0.030*x*^*2*^ + 0.0001*x*^*3*^) and saccade amplitude (*ŷ* = 14.878 + 0.723*x* − 0.012*x*^*2*^ + 0.0001*x*^*3*^), but changes in blink rate (*ŷ* = 41.780 + 0.277*x* − 0.002*x*^*2*^) had less fluctuations than in the sleep deprived condition as they were better fitted by a quadratic function. While polynomial fit did not reach significance for fixation rates in the rested condition, there was a linear increase across the driving duration (*ŷ* = 63.081 + 0.077*x*) – see Fig. 2.

**Figure 3:**
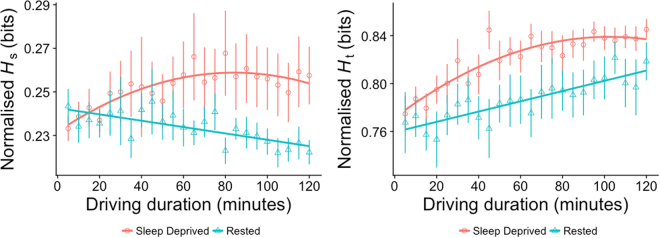
Mean and standard errors across driving duration by condition (red = sleep-deprived, green = rested) with curvilinear fits for stationary entropy - Left, sleep-deprived: (*ŷ* = 0.262 + 0.0001*x* − 0.000004*x*^*2*^), rested: (*ŷ* = 0.266 − 0.0001*x*), and gaze transition entropy - Right, sleep-deprived: (*ŷ* = 0.784 + 0.001*x* − 0.00001*x*^*2*^), rested: (*ŷ* = 0.826 + 0.0004*x*).

Changes in stationary (*F*_[21,410]_ = 41.35, *p* < 0.001, *R*^2^ = 0.66) and transition (*F*_[21,410]_ = 46.83, *p* < 0.001, *R*^2^ = 0.69) entropy of gaze across the driving duration were also better characterised by polynomial regression models – see Fig. 3. Gaze appeared to become more dispersed with time in the sleep deprived condition as *H*_*s*_ (*ŷ* = 0.262 + 0.0001*x* − 0.000004*x*^*2*^) increased with only slight reduction towards the end. However, gaze dispersion appeared to reduce in the rested condition as *H*_*s*_ (*ŷ* = 0.266 − 0.0001*x*) showed a slight but significant linear reduction with time. Visual scanning patterns also became less ordered across the driving duration in the sleep deprived condition with minimal reduction towards the end as shown by a quadratic fit of *H*_*t*_ (*ŷ* = 0.784 + 0.001*x* − 0.00001*x*^*2*^). Similar changes were observed in the rested condition, but in a more linear manner *H*_*t*_ (*ŷ* = 0.826 + 0.0004*x*)-see *Figure*. In the sleep-deprived condition, the rate of lane departure events increased by 7% with every 5-minute of driving (rate ratio = 1.065, 95% CI: 0.996, 1.127, *p* = 0.04) while in the rested condition, there was only a marginal effect of lane departure events decreasing with driving duration (*p* = 0.09) - see Fig. 1 (*Right*).

### Prediction of likelihood for lane departure event

**Figure 4:**
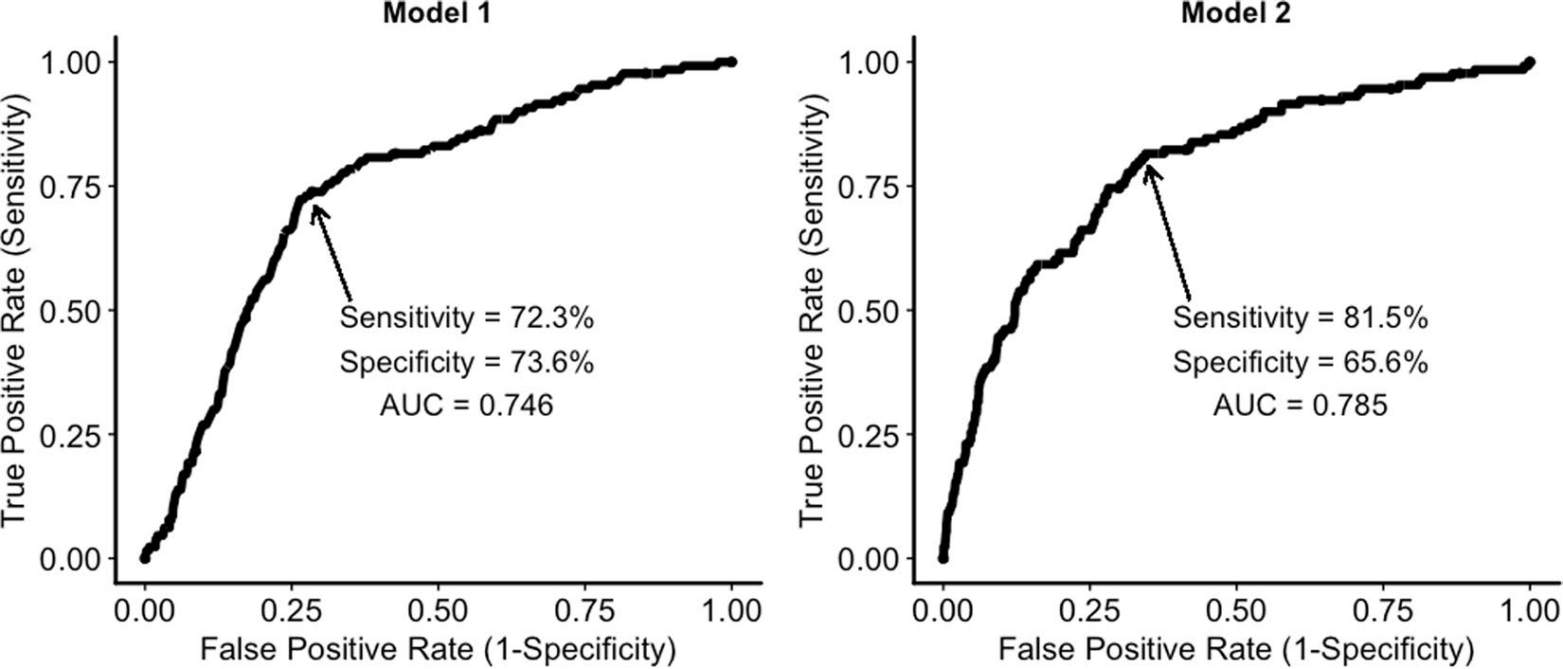
Receiver Operating Characteristic Curve for lane departure predictor model using the interaction between condition and driving duration alone (*Left*) and with addition of ocular and entropy variables (*Right*).

As lane departure events were recorded per minute, for the purpose of generating prediction models, ocular data were organised into 1-minute bins. Individual prediction of lane departure events by each of the independent variables is listed in Table 2. We constructed a base model by including condition, driving duration and their interaction as predictors which generated 72% sensitivity, 74% specificity and 75% overall accuracy as depicted by the area under the curve (AUC) in a Reciever Operating Characteristic (ROC) curve – see Fig. 4 (*Left*). This model revealed that the odds of a lane departure event occurring was three times more likely when participants were sleep-deprived (odds ratio = 3.20, 95% CI: 1.49, 7.34, *p* = 0.004) and that this likelihood increased by 1% with every minute of driving (odds ratio = 1.014, 95% CI: 1.001, 1.03, *p* = 0.04).

**Table 2:** Odds ratio, 95% CI and ROC curve properties (sensitivity, specificity and area under the curve), and significance levels for binomial models predicting lane deviation events with individual predictors.

	OR	95% CI	Sens. %	Spec. %	AUC %	*p*
Sleep deprivation	6.53	4.31, 10.21	0.79	0.64	0.71	**<0.001**
Driving duration	1.00	0.99, 1.00	0.89	0.21	0.51	0.38
Blink rate (count)	1.02	1.015, 1.03	0.36	0.85	0.66	**<0.001**
Blink duration (ms)	1.009	1.005, 1.01	0.84	0.42	0.63	**<0.001**
Fixation rate (count)	0.972	0.965, 0.98	0.57	0.74	0.69	**<0.001**
Saccade amplitude (°)	1.02	1.015, 1.03	0.56	0.73	0.65	**<0.001**
*H*_*s*_ (bits)	1.15	1.08, 1.22	0.75	0.44	0.61	**<0.001**
*H*_*t*_ (bits)	1.07	1.04, 1.10	0.59	0.83	0.62	**<0.001**

In a second model, we added all ocular measures with condition and driving duration, which led to a significant improvement of prediction (χ^2^_[6]_ = 41.07, *p* < 0.001), with 82% sensitivity, 66% specificity and 79% AUC – see Fig. 4 (*Right*). In this model, lane departure events were still three times more likely to occur in the sleep-deprived condition (odds ratio = 2.50, 95% CI: 1.16, 6.17, *p* = 0.02), but the interaction between condition and driving duration only trended towards marginal significance (*p* = 0.096). Instead, the odds increased by 7% with every 1% increase in stationary entropy (see *Gaze entropy analysis* in Methods section for details on normalisation of entropy values and their transformation for prediction), *H*_*s*_ (odds ratio = 1.07, 95% CI: 1.01, 1.14, *p* = 0.01) and reduced by 1% with every increase in fixation rate (odds ratio = 0.989, 95% CI: 0.979, 0.999, p = 0.01). No other ocular measures made significant contribution to the prediction of lane departure events in this model.

## Discussion

### Driving performance

We set out to examine how driving performance and gaze behaviour change following sleep deprivation in an on-road setting, and whether these changes are exacerbated by driving duration. Consistent with previous findings and in support of our hypothesis, the results demonstrate that sleep deprivation leads to slower reaction time^38,39^, increased number of lane departures^14^ and increased early terminations of the drive due to fatigue^16^. Following sleep deprivation, participants were three times more likely to deviate from the lane centre, and this likelihood significantly increased with amount of time driven. Similarly, the overall rate of lane departure events increased with time in the sleep-deprived condition, while no such effect was observed in the rested condition. These findings suggest that 1 night of total sleep deprivation leads to drowsy driving which impairs performance, and that it makes drivers more susceptible to task-induced fatigue which further exacerbates performance decrement.

### Gaze behaviour

As we expected, the rate and duration of blinks increased after sleep deprivation, while fixation rates reduced, suggesting an increase in the amount of time spent with eyes closed, which is a well-established effect of drowsiness^14–17^. The significant increase in saccade amplitude, which is indicative of spatial distance between fixations^40^, and stationary gaze entropy also suggest that sleep deprivation caused a more dispersed distribution of gaze, while the increase in gaze transition entropy implies a more random pattern of visual scanning. Such disruption of visual scanning behaviour suggests that top-down modulation of visual attention which aids the control of gaze distribution^41,42^ may be impaired by sleep deprivation^36,43^.

**Figure 5:**
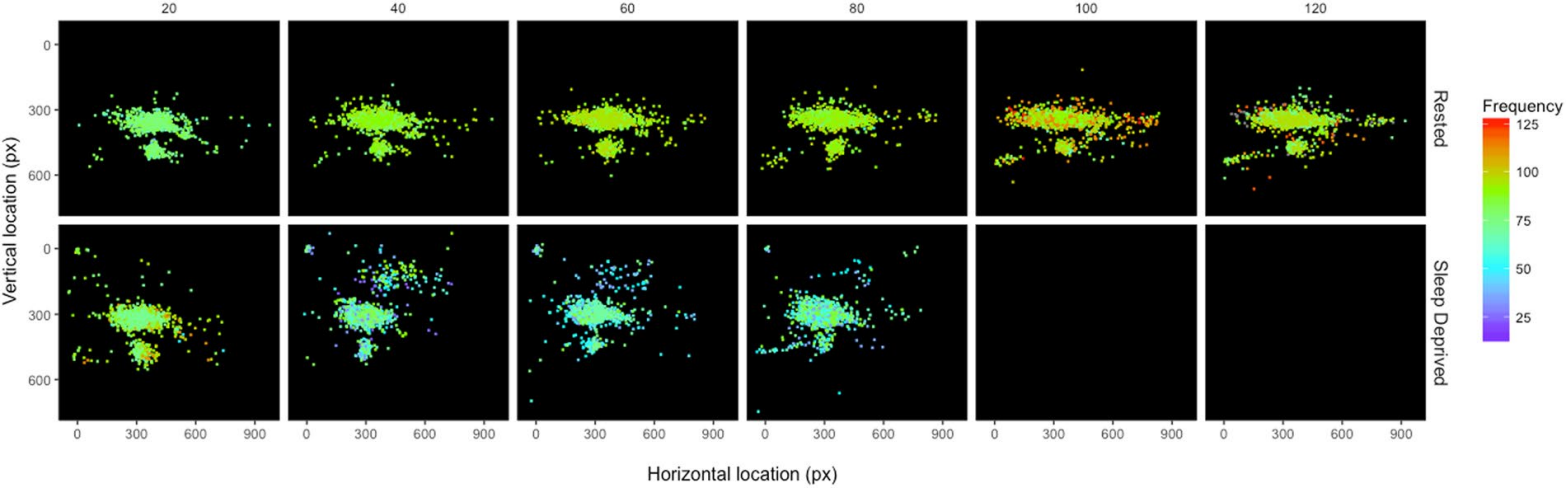
Fixation density plot for participant 6 in the rested (top row) and sleep-deprived (bottom row) conditions per 20-minute of the drive duration. Note: this participant elected to stop driving after 80 minutes in the sleep-deprived condition.

To our knowledge, the effect of sleep deprivation on gaze entropy has not previously been examined. An intuitive approach to interpret gaze behaviour and entropy in the context of driving may be to consider voluntary eye movements as a method of sampling spatially spread visual information to aid the task^44^. Thus, the reduced fixation rate in our results suggests a decline in the amount of information sampled, while the increase in stationary gaze entropy indicates a change in the spatial areas information is being sampled from, which is illustrated by the less populated and more dispersed depiction of fixation density in Fig. 5. Such change in spatial sampling associated with an increase in gaze entropy is consistent with previous findings where greater entropy was observed in relation to a more explorative than exploitative or intentional viewing behaviour^45,46^.

The increase in gaze transition entropy, on the other hand, reflects a less ordered pattern of sampling visual information over time through the transition of fixations from one area to the next. This interpretation of gaze transition entropy as a measure of disorder in scanning patterns is in agreement with a previous report where transition entropy reduced with increasing familiarity of the visual environment^47^. With respect to driving, gaze transition entropy may reflect task-related habitual sequences of glancing at specific areas of the visual field^40^, which is shown to be affected by age^25^, and according to our results, may be disrupted by sleep deprivation and task-induced fatigue to be more random than structured. Overall, these results suggest that sleep deprivation influenced visual scanning not only by reducing the rate of fixations, but also by making their locations more spatially dispersed as well as altering the pattern of transitions between fixations to be more random than what is observed in the rested condition.

The combined effect of sleep deprivation and driving duration led to greater fluctuations in fixation and blink rates over time, whereas saccade amplitude and blink duration had a similar pattern of change across both conditions. While the overall increase in these parameters may indicate the influence of task-induced fatigue^48^, the fluctuations over time are not what we expected and may partially be influenced by the rate of early termination of the driving task. The sleep-deprived condition showed a greater increase in blink rate and decrease in fixation rate over time highlighting the presence of drowsiness^49^ as a combined result of both sleep deprivation and driving duration. This effect was further reflected by the gradual increase in stationary and transition gaze entropy. The entropy results suggest that in the sleep-deprived condition gaze distribution became more dispersed and scanning patterns more random over time, while in the rested condition gaze dispersion appeared to reduce over time although the scanning patterns did become more random. Interestingly, the reduction in stationary gaze entropy for the rested condition is contrary to our hypothesis, but it appears to mirror the direction of change in the rate of lane departure events.

### Prediction of lane departure events

In terms of driving performance, our primary objective was to determine whether decrements associated with sleep deprivation were exacerbated by driving duration. Thus, we examined how well these two factors can predict the likelihood of a lane departure event occurrence. The results showed that being sleep-deprived increased the odds of lane departure events which continued to increase with every minute of driving. This predictor model showed a reasonable balance of sensitivity and specificity with overall accuracy of 75%. We were also interested in how well driving performance could be predicted by changes in gaze behaviour resulting from sleep deprivation. Thus, we examined each of the ocular and entropy variables individually and found that they made significant contributions to the prediction of lane departure events with varying degrees of accuracy – see Table 2. Blink duration and *H*_*s*_ showed the highest levels of sensitivity, while blink rate, *H*_*t*_, fixation rate and saccade amplitude had higher specificity.

To improve classification rate, we included the ocular and entropy variables to our predictor model and found that sensitivity increased by 10% and overall accuracy by 4% although specificity reduced by 8% from the previous model which only used condition and driving duration as predictors. In using such measures for the purpose of implementation into driver alerting systems, we argue that higher sensitivity (increasing true positive classification) is more important than specificity (reducing false positive classification). Thus, the model with the ocular and entropy variables is better suited for such purpose. What is interesting here is that the significant predictors in the second model, other than condition, were stationary gaze entropy and fixation rate, with contribution from driving duration only trending towards significance. As maximum driving duration in our experiment was 2 hours, and there was a high rate of early termination in the sleep-deprived condition, driving duration may have had more impact if it continued for longer than 2 hours^50^.

The results from our second model suggest that dispersion of gaze and frequency of fixations are the more likely predictors of lane departure events, which reinforces our earlier speculation that reduced fixation frequency and higher stationary gaze entropy alter the amount and spatial property of visual information sampled during driving. Since wider gaze dispersion and fewer fixations would result in reduced monitoring of the road right in front of the vehicle where one is likely to notice their position in relation to lines on the road, it is conceivable that these measures predict the likelihood of a lane departure event better than other relevant variables. Contrary to our expectation, gaze transition entropy did not make significant contribution in the prediction model. As this measure pertains more to the pattern of visual scanning, it is probable that our experimental setting and the driving performance measure used were unsuitable for utilising gaze transition entropy as a predictor. For instance, if the driving task was conducted in the presence of other traffic and intersections, gaze transition entropy may have greater relevance for more complex performance measures requiring awareness of the wider environment than just maintaining lane position (e.g. deciding when to safely turn at an intersection).

### Limitations

Although the driving task was conducted in a real vehicle, the lack of traffic in the closed circuit made it less realistic, decreasing the number of hazards and visual cues to which drivers would normally need to respond. Such environmental limitation may have reduced visuospatial complexity, thus reducing the generalisability of these results across all real-life driving situations. Additionally, participants’ performance may have been influenced by the presence of a driving instructor in the front seat, and a technician in the back to monitor the recording of gaze activity. Finally, our relatively small sample size did not allow for in-depth analysis and discussion regarding individual variance in response to seep-deprivation beyond controlling for such influence and focusing on overall effects of condition and driving duration. While our findings provide much needed insight into drivers’ visual scanning impairment as a result of sleep deprivation and fatigue-induced drowsiness, further studies are required to better understand how factors such as individual variance in circadian rhythms^51^ influence gaze entropy and its predictive power for identifying decrements in driving performance.

### Conclusion

The widespread prevalence of sleep impairment and deprivation, which leads to drowsy and fatigued driving, is a public hazard that puts not only the affected drivers, but also other road users in danger. Unlike alcohol and other drugs, there are no reliable, practical methods of testing to assess drowsiness or fatigue. Therefore, identifying key physiological indicators is a critical step towards the development of effective assessment methods and driver alerting systems to reduce fatalities associated with fatigued driving. Numerous studies using various blink parameters and electroencephalography (EEG) to monitor microsleep states have already demonstrated how sleep deprivation can impair driving performance due to time spent with eyes closed^14,15,52^. The present study provides new evidence to reveal the less readily observable impact of drowsiness, and demonstrates how visual perception following sleep deprivation is not only affected by slow blinks and microsleep, but also by deterioration of top-down gaze control to alter the frequency, spatial distribution and pattern of scanning through which visual information is sampled.

Given that visual scanning appears to be an important determinant of crash risk^13^, these findings have important implications for the development of driver drowsiness detection methods to reduce road accidents. With recent advancements in vehicle technology, which allow simultaneous monitoring of driver state and the environment^53^, integrating gaze entropy analysis into such systems may allow for a more precise alerting system by matching driver state to visual demands of the environment. Future studies should examine how the influence of sleep deprivation on gaze entropy may alter depending on complexity of visual environment and task demand to better understand and determine an optimal range of gaze entropy to safely execute a driving task under varied environmental conditions.

## Methods

### Participants

Nine participants (4 male, mean age: 33 ± 7.07, 5 female, mean age: 34 ± 10.22) were recruited through community advertising. All participants held valid Australian or international driver license with a minimum of 3 years driving experience, and had normal vision. Participants were screened for any chronic neurological, cardiac or respiratory illnesses; sleep disorders and use of drugs or sedative medications. All participants were within the average range (<10) of daytime sleepiness as measured by the Epworth Sleepiness Scale (ESS)^54^, and were assessed as having low to moderate habitual consumption of caffeine (<300 mg per day) and alcohol (<5 standard drinks per week). Participants provided written informed consent and were compensated for their time at the end of the study. Ethical approval was obtained from the Austin Health Human Research Ethics Committee.

### Procedure

Each participant completed two 2-hour driving sessions (1 after a normal night of sleep, 1 following a night of sleep deprivation) in a randomised order, with one-week interval between sessions. For 7 days prior to each driving session, participants were instructed to maintain a fixed sleep-wake schedule of 8 hours in bed from 23:00 h to 07:00 h, which was monitored by wrist actigraphy (*Actiwatch*, Philips Respironics Ltd). The night prior to driving in the sleep-deprived condition, participants stayed at the Austin Sleep Clinic, Melbourne, where they were monitored by staff to ensure they remain awake. All driving sessions were conducted between the hours of 2–5 pm, in an instrumented automatic-transmission vehicle on a closed track. A qualified instructor who was blinded to the conditions of the study accompanied each participant to observe their performance and intervene if they were at risk of collision, while a technician sat in the back to monitor the recording of saccade activity.

Prior to each driving session, participants completed a visual psychomotor vigilance task^55^ (PVT) and the Karolinska Sleepiness Scale (KSS) questionnaire^56^, after which they sat in the vehicle and were fitted with the eye tracking system. A cap-mounted eye tracking system (SensoMotoric Instruments, Teltow, Germany) was used to collect on-line gaze tracking data during the drive. The SMI equipment is a video based mobile eye tracking system with eye and scene cameras, connected to a laptop with iView (recording) and BeGaze (analysis) software^57^. A 5-point calibration process was undertaken before each drive.

### Data

Ocular activity was recorded at 50 Hz frequency rate and events filtered using the built-in dispersion-based event detection algorithm within SMI’s BeGaze analysis software^57^, with 80 ms minimum duration and 100 px maximum dispersion thresholds. To reduce the influence of microsleep^52^ in our analysis, blink events were filtered as those with duration $\le$300 ms. Lane departure events were recorded as counts per minute.

### Gaze entropy analysis

Stationary gaze entropy analysis was conducted using Shannon’s entropy equation^30^ expressed as:

1 $${H}_{s}(x)=\,-\sum _{i=1}^{N}\,p(i).lo{g}_{2}p(i),$$

where *H*_*s*_ is the entropy value of set *x* (a time bin for each participant in each condition), _*i*_ represents state spaces or the location (coordinates in 2D plane) of each fixation contained in *x*, *N* is the total number of fixations within *x*, and *p* is the proportion of fixations landing on a given state space within *x*. Gaze transition entropy was conducted by applying the conditional entropy equation to 1^st^ order Markov transitions of fixations^32^ as follows:

2 $${H}_{c}(x)=-\,\sum _{i=1}^{N}p(i)[\sum _{j=1}^{N}p(i|j).lo{g}_{2}p(i|j)],\,i\ne j,$$

where *p*(*i*) is the stationary distribution of fixation locations, *p*(*i| j*) is the probability of transitioning to *j* given current position of *i*, and *i ≠ j* denotes exclusion of transitions within the same state space from the inner summation – see Ellis & Stark^33^ for detailed explanation on the application of this equation to gaze data.

To apply these equations for the purpose of condition alone and condition by driving duration analysis, fixation coordinates were discretised by organising them into spatial bins of 100 × 100 pixel. This allowed for generation of state spaces across the visual field with sufficient transition distributions. Dividing the visual field in this manner was suitable for examining the overall distribution of gaze and transition patterns across the entire driving duration and 5-minute time bins. However, visual scanning for the purpose of maintaining lane position requires a more centralised distribution of gaze. Therefore, spatial precision was increased by creating 30 × 30 pixel spatial bins when calculating entropy in 1-minute time bins for predicting lane departure events. Due to the logarithm of 0 being undefined, state spaces that were not occupied by at least one fixation were excluded.

For interpretability, the entropy values were normalised through division by maximum possible entropy (*H*_*max*_). Maximum entropy, which is equal to logarithm (base 2) of the number of state spaces, occurs when the distribution of information is equal across all state spaces^30^; or in the case of this study, when the proportion of fixations landing on each of the occupied spatial bins within a given time-bin are equal. For transition entropy, *H*_*max*_ is equal to the logarithm of the number of valid transitions between state spaces. The resulting normalised value for both stationary and transition entropy (range 0–1) can be interpreted as a percentage of the maximum possible entropy for fixations generated in a given time-bin. For the purpose of predicting lane departure events, these values were transformed through multiplication by 100 in order to calculate odds ratio per 1% increase in entropy values.

### Missing data

Some participants elected to terminate the drive earlier in the sleep-deprived condition, which resulted in some missing data. As these early terminations were a result of condition effect and the data were not missing at random; all data were organised into 5-minute bins and missing data imputed by carrying the mean of last observed 5-minute bin forward^58^ for the purpose of condition by time analysis. This resulted in total observations of 432 (24 time bins × 9 participants × 2 conditions).

### Statistical analysis

All statistical analyses were conducted in R 3.3.0^59^. Paired t-tests were conducted to examine the effect of condition on PVT and KSS measures. Repeated measures analysis of variance (ANOVA) with fixed effects was used to test the effect of condition on blink and fixation parameters. Poisson regression was conducted to test the effects of condition and time on rates of lane departure events and a Kaplan-Meier survival curve fitted to compare probability to continue driving in each group. To examine the combined effects of condition and driving duration, polynomial mixed models were applied. And finally, binomial logistic regression was used to predict the odds of a lane departure event occurring. Predictor models were further examined for their sensitivity, specificity and overall accuracy in predicting lane departure by plotting ROC curves^60^.

### Significance

Eye-monitoring technology is being implemented in motor vehicle manufacturing with the intention of alerting the driver and, in autonomous vehicles, initiating control transfer to the vehicle should the driver be impaired. Our findings show that gaze entropy measures have the potential to be an effective mode of monitoring driver-state during periods of open-eyed visual scanning to detect potential risk of impairment in driving performance.
